# Serum macrophage inhibitory cytokine‐1 as a clinical marker for non–small cell lung cancer

**DOI:** 10.1111/jcmm.16360

**Published:** 2021-02-18

**Authors:** Chunhua Xu, Li Li, Wei Wang, Qian Zhang, Xiuwei Zhang, Rusong Yang

**Affiliations:** ^1^ Department of Respiratory Medicine Nanjing Chest Hospital Nanjing China; ^2^ Affiliated Nanjing Brain Hospital Nanjing Medical University Nanjing China; ^3^ Department of Respiratory Medicine Affiliated Jiangning Hospital of Nanjing Medical University Nanjing China; ^4^ Department of Thoracic Surgery Nanjing Chest Hospital Nanjing China

**Keywords:** diagnosis, macrophage inhibitory cytokine‐1, non–small cell lung cancer, prognosis

## Abstract

The aim of this study was to investigate the value of serum macrophage inhibitory factor‐1 (MIC‐1) level in patients with non–small cell lung cancer (NSCLC). Serum samples from 296 patients with NSCLC and 240 healthy controls were collected. The levels of serum MIC‐1 were determined by ELISA. The serum MIC‐1 levels in NSCLC patients were higher than that of the controls (*P* <.001). Univariate and multivariate Cox regression analysis showed that serum MIC‐1 was an independent prognostic indicator of OS and PFS. Serum MIC‐1 is a valuable biomarker for the diagnosis and prognosis of NSCLC.

## 1 INTRODUCTION

Lung cancer is the main cause of cancer‐related death in the world.^1^ More than 75% of lung cancer patients are diagnosed as advanced disease, which makes the 5‐year survival rate less than 15%.^2^ The disadvantage of carcinoembryonic antigen (CEA) is poor sensitivity and specificity in the diagnosis of early lung cancer.^3^ Therefore, more research is needed to find new biomarkers in order to diagnose and predict the progress of lung cancer.

Macrophage inhibitory cytokine‐1 (MIC‐1) is a secretory protein of the transforming growth factor‐β family and is involved in carcinogenesis‐related processes, including proliferation, migration, apoptosis and angiogenesis.^4^, ^5^, ^6^ Previous studies have found the value of serum MIC‐1 level in the diagnosis of colorectal cancer, prostate cancer, pancreatic cancer and so on.^7^, ^8^, ^9^, ^10^ Recently, it has been reported that serum MIC‐1 may be a potential biomarker in NSCLC.^11^, ^12^ However, the relationship between serum MIC‐1 and the progression of NSCLC and the effect of MIC‐1 on the NSCLC survival have not been fully evaluated.

In this study, we investigated the relationship between serum MIC‐1 and clinicopathological features and patients' survival. The results showed that serum MIC‐1 could be used as a biomarker of diagnosis and prognosis of NSCLC.

## 2 METHODS

### 2.1 Patients

This was a prospective trial. 296 NSCLC patients (aged 26‐77 years) and 240 gender and age‐matched healthy controls (aged 37‐68 years) were recruited. The patient data were collected, including age, gender, smoking, histological type, grade, stage and outcome. Follow‐up information is obtained through telephone survey or WeChat. The last follow‐up was on 20 February 2019. Progression‐free survival (PFS) was defined as the time interval between the date of diagnosis and the date of recurrence. Overall survival (OS) was defined as the time interval between the date of diagnosis and the date of death.

The study protocol was approved by the Ethics Committee of the Nanjing Chest Hospital. All patients provided written informed consent before enrolment.

### 2.2 Measurement of serum MIC‐1 and CEA levels

Serum samples were taken from each person prior to the start of the treatment. The sensitive internal sandwich ELISA was used to detect the serum MIC‐1 levels. The CEA levels were measured by electrochemiluminescence immunoassay on Roche Elecsys 1010 Analyzer (Roche Diagnostics). All the samples were ignored by the technicians running the tests.

### 2.3 Statistical analysis

Statistical software (SPSS for Windows, version 18) was used for the analysis. The Mann‐Whitney *U* test was used to determine the difference between the two groups. The cut‐off value of the serum concentrations of parameters was calculated using a receiver operating characteristic (ROC) curve. Univariate analysis was performed using the Kaplan‐Meier method and the log‐rank test. Multivariate analysis was conducted to determine an independent impact on survival using the Cox proportional hazard method. *P* <.05 was considered statistically significant.

## 3 RESULTS

### 3.1 Serum levels of MIC‐1 and CEA in NSCLC patients and healthy controls

The serum levels of MIC‐1 in NSCLC patients were higher than those of the controls (1582.31 ± 473.01 pg/mL vs 507.71 ± 107.64 pg/mL, *P* <.001). The serum CEA levels of NSCLC patients were also higher than those of the controls (29.78 ± 7.71 ng/mL vs 3.36 ± 1.25ng/mL, *P* <.001).

### 3.2 Diagnostic value of MIC‐1 and CEA in NSCLC patients

**FIGURE 1:**
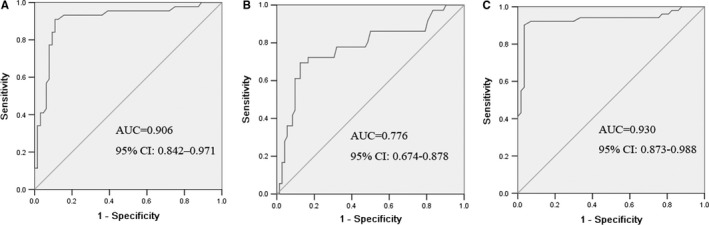
ROC curves for the serum MIC‐1 (A) and CEA (B) and MIC‐1 + CEA (C) in differentiating NSCLC patients and healthy controls. The areas under the curve of serum MIC‐1, CEA and MIC‐1 + CEA were 0.906, 0.776 and 0.930, respectively

The ROC curve was used to calculate the sensitivity of the marker in separating NSCLC patients from healthy controls. As shown in Figure 1A, an area under the curve (AUC) value for serum MIC‐1 reached 0.906 (confidence interval (95% CI): 0.842‐0.971). With a cut‐off value of 1000 pg/mL, the sensitivity, specificity, accuracy, positive predictive value and negative predictive value of serum MIC‐1 were 63.5%, 95.0%, 77.6%, 94.0% and 67.9%, respectively. These results indicated that serum MIC‐1 is a valuable biomarker for the diagnosis of NSCLC.

Detection of CEA and analysis of its diagnostic value were analysed. The area under the CEA ROC was 0.776. It was lower compared with the areas of MIC‐1 (Figure 1B). With a cut‐off value of 5.0 ng/mL, CEA had a sensitivity of 47.3%, a specificity of 93.3%, an accuracy of 67.9%, a positive predictive value of 89.7% and a negative predictive value of 58.9%. The sensitivity of MIC‐1 was higher compared with CEA.

The diagnostic value of MIC‐1 combined with CEA in NSCLC was further analysed. The results showed that the combined detection of these two indices had a sensitivity of 77.0% and a specificity of 95.8%. The combination of MIC‐1 and CEA had better sensitivity and specificity than MIC‐1 and CEA alone (Figure 1C).

### 3.3 Association between MIC‐1 levels and clinicopathological characteristics

The relationship between serum MIC‐1 levels and clinicopathological characteristics of lung cancer was analysed. MIC‐1 levels were correlated with TNM stage (*P* =.001), tumour differentiation (*P* =.001) and lymph node metastasis (*P* =.004).

### 3.4 Prognostic value of serum MIC‐1 levels for NSCLC patients

**FIGURE 2:**
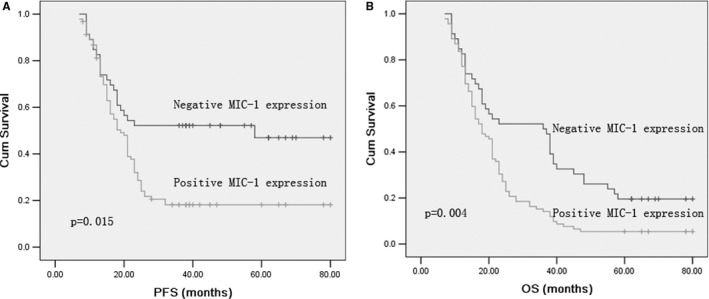
Kaplan–Meier survival curves for PFS and OS in patients with MIC‐1–positive and MIC‐1–negative NSCLC. Log‐rank test determined that the PFS (A) and OS (B) in positive MIC‐1 patients were significantly shorter than those in the negative MIC‐1 patients (*P* =.015, *P* =.004)

Univariate analysis showed that serum MIC‐1 levels were correlated with OS (*P* =.005) and PFS (*P* =.004, Table 1). In multivariate analysis, MIC‐1–positive was significantly correlated with shorter PFS and OS (*P* =.002 and *P* =.007). The Kaplan‐Meier survival curve further confirmed that PFS and OS of NSCLC patients with MIC‐1–positive were significantly shorter than those of NSCLC patients with MIC‐1–negative (Figure 2).

**TABLE 1:** Univariate and multivariate Cox analysis of variables considered for PFS and OS of NSCLC patients

Characteristics	Univariate	Multivariate	HR (95% CI)	*P*
	HR (95% CI)	*P*		
PFS				
Gender (male vs female)	1.175(0.694‐1.990)	.549	0.987(0.844‐1.15)	.866
Age (<60 vs ≥60)	0.887(0.722‐1.090)	.0.255	0.689(0.351‐1.35)	.279
Histological type (SCC vs ADC)	0.721(0.385‐1.347)	.0.305	0.822(0.353‐1.91)	.650
Differentiation (well‐moderate vs poor)	0.671(0.346‐1.320)	.0.238	1.134(0.483‐2.66)	.773
TNM stage (I‐II vs III‐IV)	1.016(1.002‐1.029)	.00.021*	1.834(1.053‐3.19)	.032*
Lymph node metastasis (N_0_ vs N_1‐3_)	1.940(0.767‐4.909)	.0.162	1.316(0.838‐2.06)	.233
MIC‐1 (negative vs positive)	2.230(1.288‐3.860)	.00.004*	2.881(1.460‐5.68)	.002*
OS				
Gender (male vs female)	1.016(0.570‐1.812)	.957	0.755(0.390‐1.46)	.404
Age (<60 vs ≥60)	1.044(0.474‐2.302)	.914	1.270(0.636‐2.53)	.498
Histological type (SCC vs ADC)	0.690(0.245‐1.943)	.483	2.107(0.731‐6.60)	.167
Differentiation (well‐moderate vs poor)	1.544(0.640‐3.727)	.334	1.004(0.606‐1.66)	.987
TNM stage (I‐II vs III‐IV)	1.303(1.261‐1.346)	.001*	1.321(1.278‐1.36)	.001*
Lymph node metastasis (N_0_ vs N_1‐3_)	1.904(0.627‐5.780)	.256	1.013(0.366‐2.80)	.980
MIC‐1 (negative vs positive)	2.425(1.314‐4.475)	.005*	2.247(1.246‐4.05)	.007*

Abbreviations: ADC, adenocarcinoma; CI, confidence interval; HR, hazard ratio; OS, overall survival; PFS, progression‐free survival; SCC, squamous cell carcinoma.

* Statistically significant difference (*P* <.05)

## 4 DISCUSSION

Some studies have shown that MIC‐1 can be used as a diagnostic marker for some types of tumours.^7^, ^8^, ^9^, ^10^, ^11^ However, the value of serum MIC‐1 level in the diagnosis and prognosis of NSCLC has not been fully elucidated. In this study, the levels of MIC‐1 in NSCLC were higher than those in healthy controls. The diagnostic sensitivity and specificity of MIC‐1 were 63.5% and 95.0% in NSCLC patients. The results showed that MIC‐1 was valuable in the diagnosis of NSCLC. In addition, we found that the levels of MIC‐1 were significantly correlated with lymph node metastasis, tumour differentiation and TNM stage, suggesting that MIC‐1 may be an indicator of tumour progression in NSCLC patients.

To further analyse the diagnostic value of MIC‐1 combined with CEA in NSCLC. The results showed that the combination of MIC‐1 and CEA has better diagnostic value than the single index. This may provide a new method for the diagnosis of NSCLC.

Previous studies have shown that the expression of MIC‐1 is related to the prognosis of lung cancer.^11^, ^12^ Our study showed that MIC‐1–positive was significantly related to the decrease in PFS and OS. The Kaplan‐Meier survival curve further illustrates this relationship. It is suggested that the determination of serum MIC‐1 level is helpful to predict the prognosis of NSCLC patients.

Several limitations of our study warrant discussion. First, we performed the study at a single centre with relatively small sample size. Second, the expression of MIC‐1 in serum of lung cancer patients was detected, but the expression of MIC‐1 in lung cancer tissues was not detected. Third, the specific mechanism of the relationship between MIC‐1 expression and NSCLC was lacking. Further perspective trial should be performed.

In conclusion, our results suggest that serum MIC‐1 may be a valuable biomarker for the diagnosis and prognosis of NSCLC.

## CONFLICT OF INTEREST

The authors declare no conflicts of interest in this work.

## AUTHOR CONTRIBUTIONS

**Chunhua Xu:** Conceptualization (equal); Writing‐original draft (equal). **Li Li:** Methodology (equal); Writing‐original draft (equal). **Wei Wang:** Methodology (equal). **Qian Zhang:** Formal analysis (equal); Resources (equal). **Xiuwei Zhang:** Conceptualization (equal). **Rusong Yang:** Methodology (supporting).

## Data Availability

All data generated or analysed during this study are included in this article.
